# A new measure for multi-professional medical team communication: design and methodology for multilingual measurement development

**DOI:** 10.3389/fped.2023.1127633

**Published:** 2023-06-02

**Authors:** Sara Malone, Jocelyn Rivera, Maria Puerto-Torres, Kim Prewitt, Firas Sakaan, Lara Counts, Zebin Al Zebin, Anita V. Arias, Parthasarathi Bhattacharyya, Sanjeeva Gunasekera, Sherry Johnson, Joyce Kambugu, Erica C. Kaye, Belinda Mandrell, Jennifer W. Mack, Jennifer McArthur, Alejandra Mendez, Lisa Morrissey, Rana Sharara-Chami, Jennifer Snaman, Elizabeth Sniderman, Douglas A. Luke, Dylan E. Graetz, Asya Agulnik

**Affiliations:** ^1^ Department of Surgery, Washington University in St. Louis School of Medicine, St. Louis, MO, United States; ^2^ Department of Pediatrics, Hospital Infantil Teletón de Oncologia (HITO), Querétaro, Mexico; ^3^ Division of Critical Care Medicine, Department of Global Pediatric Medicine, St. Jude Children’s Research Hospital, Memphis, TN, United States; ^4^ Center for Public Health Systems Science, Washington University, St. Louis, MO, USA; ^5^ Pediatric Hematology and Oncology, King Hussein Cancer Center, Amman, Jordan; ^6^ Department of Pediatric Oncology Critical Care, Tata Medical Center, Kolkata, India; ^7^ National Cancer Institute, Maharagama, Sri Lanka; ^8^ Pediatric Oncology, Uganda Cancer Institute, Kampala, Uganda; ^9^ Department of Hematology and Oncology, Dana-Farber Cancer Institute and Boston Children’s Hospital, Boston, MA, United States; ^10^ Pediatric Intensive Care Unit, Unidad Nacional de Oncologia Pediatrica (UNOP), Guatemala City, Guatemala; ^11^ Pediatric Critical Care Medicine, American University of Beirut, Beirut, Lebanon; ^12^ Pediatric Intensive Care Unit, LJ Murphy Inova Children’s Hospital, Fairfax, VA, United States; ^13^ Northern Alberta Children's Cancer Program, Stollery Children's Hospital, Edmonton, AB, Canada

**Keywords:** measurement, implementation science, communication, health equity, bilingual

## Abstract

**Background:**

As implementation science in global health continues to evolve, there is a need for valid and reliable measures that consider diverse linguistic and cultural contexts. A standardized, reproducible process for multilingual measure development may improve accessibility and validity by participants in global health settings. To address this need, we propose a rigorous methodology for multilingual measurement development. We use the example of a novel measure of multi-professional team communication quality, a determinant of implementation efforts.

**Methods:**

The development and translation of this novel bilingual measure is comprised of seven steps. In this paper, we describe a measure developed in English and Spanish, however, this approach is not language specific. Participants are engaged throughout the process: first, an interprofessional panel of experts and second, through cognitive interviewing for measure refinement. The steps of measure development included: (1) literature review to identify previous measures of team communication; (2) development of an initial measure by the expert panel; (3) cognitive interviewing in a phased approach with the first language (English); (4): formal, forward-backward translation process with attention to colloquialisms and regional differences in languages; (5) cognitive interviewing repeated in the second language (Spanish); (6) language synthesis to refine both instruments and unify feedback; and (7) final review of the refined measure by the expert panel.

**Results:**

A draft measure to assess quality of multi-professional team communication was developed in Spanish and English, consisting of 52 questions in 7 domains. This measure is now ready for psychometric testing.

**Conclusions:**

This seven-step, rigorous process of multilingual measure development can be used in a variety of linguistic and resource settings. This method ensures development of valid and reliable tools to collect data from a wide range of participants, including those who have historically been excluded due to language barriers. Use of this method will increase both rigor and accessibility of measurement in implementation science and advance equity in research and practice.

## Background

Implementation science is becoming increasingly used in global health (1, 2). As a result, implementation research efforts must ask how current implementation science theories, measures, and models can, or cannot, be applied to global settings (2). For example, some frameworks, such as the Consolidated Framework for Implementation Research requires extension for application in low- and middle-income countries (3). There is a need to develop reliable and pragmatic measures of implementation determinants, processes, and outcomes that can be utilized across these diverse cultural and linguistic contexts.

Measurement of constructs and outcomes has been recognized as important for research in implementation science and to assist practitioners outside of the research context (4). Implementation measures are often developed for single studies and may lack rigorous evaluation to ensure reliability and validity (5, 6), limiting comparison between studies and resulting in multiple measures that have not been tested in different settings. Prior outlines of instrument development have not detailed the processes by which we should pay attention to cultural and linguistic needs within this process (7). Additionally, most measures have limited global applicability and are only useful in certain settings due to measure development in a high-income context. To advance implementation research, it is vital to construct measures that are pragmatic and valid in a variety of settings (4).

Similarly, few tools have been designed or evaluated for use in multiple languages. Existing multilingual measures have not been co-developed, but instead translated in multiple iterations without a standardized process. For example, three commonly used brief measures of implementation outcomes have been translated only after initial development and testing (8, 9). While this strategy is valid, it adds additional work and does not allow for either simultaneous refinement of both measures or up-front research in multiple linguistic settings. Translation without appropriate systematic consideration of linguistic and cultural differences can result in measures that are assessing different concepts, potentially limiting knowledge accumulation across global settings. Finally, translation after development of the initial instrument risks creation of an instrument that does not include all relevant constructs across different cultures. There is also currently no standard process to guide measure translation and reporting, which may have implications for measure quality.

To address current gaps in guidance for development of high-quality measures for use in research and practice, this paper does two things. First, we outline a 7-step method for multilingual measure development and subsequent translation. Second, we describe the use of this method through the development of a new measure that assesses the quality of multi-professional team communication in clinical settings.

This 7-step method allows for rigorous development of new measures that are useful in a variety of contexts and have linguistic validity (10, 11), which should then be refined through psychometric testing. This method has two main benefits for the field of implementation science: first, it allows for research in multiple settings that do not share a primary language, and second, it helps develop consistent and clear definitions for implementation science constructs. Ultimately, this approach allows constructs to be compared across studies and settings. Additionally, this paper provides guidance for measure translation, which represents best practices even for measures that are not being co-developed.

### Measuring team communication quality in childhood cancer care

The measure described in this study was developed to assess multi-professional communication quality within the context of pediatric oncology. Hospitalized pediatric oncology patients are at high risk of clinical deterioration (12, 13) and require excellent interdisciplinary communication to provide high-quality care. During clinical patient deteriorations, team members must alert one another to concerns, come together to consider etiologies and strategize about medical interventions, and then implement interventions, all in a coordinated and rapid manner. Previous studies have reported that effective interdisciplinary communication is a fundamental component of quality care and a priority to improve outcomes in pediatric oncology (14) and intensive care (15–17). Effective communication between clinical teams improves clinical outcomes of hospitalized patients (18), whereas lack of effective team communication contributes to a delayed response to critically ill patients (19, 20). Additionally, multi-professional team communication and processes have been identified as important determinants of implementation and sustainment of evidence-based care (3, 21, 22).

Multiple studies have demonstrated the importance of high-quality interdisciplinary communication in childhood cancer care globally, where hospitals face variability in resources to provide acute medical care– specifically, staff, equipment, and systems of care (23). However, there is currently no reliable and validated measure of the quality of interdisciplinary (different medical specialties) and multi-professional (different professions, like nurses and physicians) communication applicable to clinical settings of variable resource-levels. While there is a clear need for effective interventions to optimize interdisciplinary communication to improve patient care (24, 25), lack of reliable and valid assessment tools makes development and evaluation of such interventions challenging, especially across different practice settings. There is an urgent need for a measurement tool that provides a reliable assessment of communication and collaboration quality in clinical settings to improve patient care globally (25).

## Method

### Participants

#### Expert panel

To develop CritCom, an expert panel was assembled, including 21 experts from 9 countries with expertise in pediatric oncology medicine, critical care medicine, interdisciplinary communication, and measure development. These individuals represented different disciplines associated with critical care communication, global regions, economic and cultural backgrounds (Table 1). Panelists assisted with the development and refinement of the CritCom measure based on the literature research and their expertise in measurement, clinical care, and/or communication. This process was conducted in English and Spanish. While this could be conducted in any language, these two were selected because of its relationship to our previous work primarily focused in Latin America (14, 26).

**Table 1:** Expert panel demographics (*n* = 21).

Characteristic	*n*	%
**Discipline**		
Pediatric hematology-oncology	7	33.3%
Pediatric critical care	5	23.8%
Implementation science	3	14.3%
Pediatric hematology-oncology and palliative care	2	9.5%
Pediatric hematology-oncology and critical care	2	9.5%
Pediatric emergency medicine	1	4.8%
Biostatistics	1	4.8%
**Profession**		
Physician	12	57.1%
Nurse	5	23.8%
Social worker	1	4.8%
Non-clinical	3	14.3%
**Country**		
**HIC**		
United States	13	61.9%
**UMIC**		
Jordan	1	4.8%
Mexico	1	4.8%
Guatemala	1	4.8%
**LMIC**		
India	1	4.8%
Pakistan	1	4.8%
Lebanon	1	4.8%
**LIC**		
Uganda	1	4.8%
Zambia	1	4.8%

#### Interview participants

After an initial measure was drafted, cognitive interviews (detailed below) were conducted in both English and Spanish. Participants were recruited via an email to a wide network of global health workers to voluntarily participate in virtual interviews to analyze the preliminary survey, emphasizing its objective related to interdisciplinary communication. Thirty-six individuals from 15 countries participated in cognitive interviews. These individuals were clinicians in Pediatric Oncology or Critical Care Medicine and included both nurses and physicians practicing in hospitals with a variety of resource-levels (Table 2).

**Table 2:** Demographics for cognitive interviews (*n* = 36).

Characteristic	*n*	%
**Language**		
English	19	52.8%
Spanish	17	47.2%
**Discipline**		
Pediatric hematology-oncology	17	47.2%
Pediatric critical care	19	52.8%
**Profession**		
Physician	25	69.4%
Nurse (bedside)	9	25.0%
Nurse (management)	2	5.6%
**Country**		
**HIC**		
Chile	2	5.6%
Netherlands	2	5.6%
Spain	1	2.8%
**UMIC**		
Brazil	5	13.9%
Colombia	2	5.6%
Ecuador	2	5.6%
Guatemala	2	5.6%
Mexico	8	22.2%
Russia	2	5.6%
**LMIC**		
Egypt	1	2.8%
India	1	2.8%
Pakistan	5	13.9%
Vietnam	1	2.8%
**LIC**		0.0%
Ethiopia	1	2.8%
Sierra Leone	1	2.8%

### Procedures

**Figure 1:**
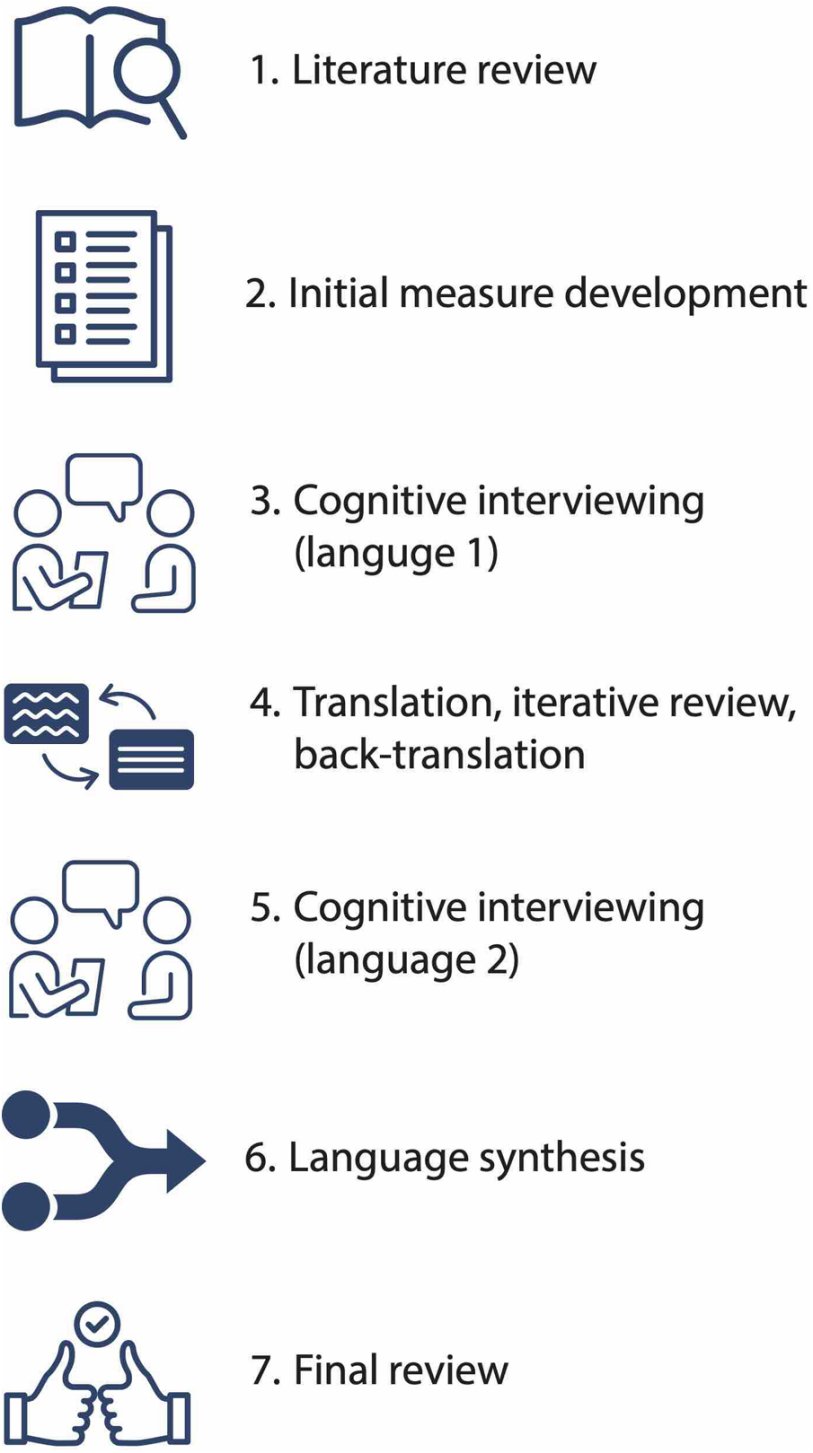
Seven step process for bilingual measurement development methods.

Below are the seven steps we recommend for multilingual measurement development (Figure 1). Additionally, we describe how these steps were operationalized for CritCom measurement development.

Step 1: literature review

A thorough literature review can help ensure that there is a need for a new measurement tool by offering a comprehensive understanding of what is available in a specific area. Additionally, a comprehensive and systematic literature review can be used for preliminary item identification.

To develop CritCom, we conducted a literature search to identify existing tools assessing teamwork and communication, focusing on quantitative measurement articles. The concepts were searched using the keywords “interdisciplinary communication,” “interprofessional communication,” “nurse-doctor communication,” “evaluation of teamwork,” and “communication in critical care.” This literature search failed to identify a single measure of quality of interdisciplinary communication around patient deterioration; however, it identified multiple studies that informed our subsequent measure development process (Table 3). Measurement tools identified by this search were used to build a database of question items and domains which were reviewed by the expert panel to verify that they address all relevant aspects of team communication.

Step 2: initial measure development

**Table 3:** Existing tools and domains.

Survey	Domains	# Items	Reported findings	Items used in CritCom
Safety Attitudes SAQ short form (38)	Teamwork climate Safety climate Job satisfaction Stress recognition Perception of management Working conditions	36	Healthcare organizations can use the survey to assess caregiver attitudes about six patient's safety-related domains, to compare themselves with other organizations, prompt interventions to improve safety attitudes, and measure the effectiveness of these interventions	Items identified as team climate domains and isolated questions related to collaboration.
SOPS® Hospital Survey Version (39)	Communication Hospital management support Leader support for patient safety Information exchange Organization Patient safety rating Teamwork Team empowerment	37	The use of the tool needs to be sensitive to the demands of every care settings, the target population, and other aspects of healthcare contexts. There is a need to develop guidelines for using, adapting, and translating the survey and reporting findings based on its use.	Items classified as team empowerment and communication were the only ones selected for the same domain.
COACH (40)	Resources Monitoring Sources for information Commitment to work Work culture Leadership Payment	50	The tool will allow for systematic description of the local healthcare context before implementing interventions to allow for modifying implementation strategies or as part of the evaluation and allow for deeper insights into the process of implementing evidence-based practices in LMICS	Questions related to work culture were selected for evaluation for empowerment.
CTEF Tool (41)	Mission framework Workload Organization Leadership Team members Task focused behaviors Task outcomes Communication	68	Allows investigating subcomponents of team effectiveness in surgical settings. Measure outcomes based on two categories—task outcomes and team outcomes. Team-related products measure team satisfaction, team norms, roles, communication patterns, motivation, attitudes, emotional tone, and turnover.	None of the questions were selected for the project on final revision because they were not related to communication around a deteriorated patient.
TeamSTEPPS (42)	Leadership Situation monitoring Team support Communication	26	Survey related to an evidence-based patient safety-training program designed to improve communication and teamwork among health care professionals by promoting a culture of team-driven care. The program creates interdisciplinary team training systems to serve as the foundation for a patient safety strategy.	Items identified as leadership were considered helpful for the hierarchy domain. Other items related to team support and some others not classified will be used as communication quality.
Ship Management Attitudes Questionnaire (43)	Teamwork climate Hierarchy Organization Management Work goals	125	Assess the attitudes of junior staff and determine how these attitudes correlate with behavior and performance in exercise. The attitude–performance linkage is not linear.	Questions classified as teamwork climate were selected for the hierarchy domain
ICU Nurse Questionnaire (44)	Collaboration *Communication quality* Leadership Perceived effectiveness Safety culture	60	Provide specific feedback on each of the measures of interest. The results can evaluate the team's performance and serve as a foundation for improving the quality of care.	Questions focused on relationships and communications within the ICU were selected as collaboration and communication quality evaluation. Questions from the leadership and safety culture were chosen as an evaluation for hierarchy. These questions will need a language adjustment applicable for the entire team, not only for the nurse staff.
IDC Survey (45)	Trigger Teamwork climate *Communication quality* *Collaboration*	33	The study focused on provider satisfaction and perception of the quality of communication and care.	Questions related to methods of communication were selected as trigger domains. The questions adapted from SAQ have been chosen from the original tool. Items from the teamwork climate were selected to be used within the collaboration domain
HITV Tool (46)	Support structures Communication Engagement and empowerment Patient care Patient care Support structures Collaboration	10	Contribute to better management practices and advancing knowledge to promote retention of nurses and other healthcare professionals and transform the acute healthcare work environment.	Most of the items were selected to evaluate communication, empowerment, and collaboration.
IPA Tool (47)	Communication Respect Altruism and caring Excellence Ethics Accountability	26	The tool measures observable behaviors of healthcare staff in training that demonstrate professionalism and cooperation when working with other healthcare providers. It was designed to be used with learners who completed their final practice experiences before attaining their professional degrees.	Most of the items were selected for communication, hierarchy, and collaboration domains. The questions classified as accountability, ethics, and altruism were not selected.

The literature review in step 1 serves to identify if there is prior work that can inform measure development. If so, these existing measures can be incorporated as appropriate. However, for content areas where there is no prior work, other strategies should be considered for rigorous measurement development. For example, concept mapping has been used as a valid strategy for item and domain generation (27).

To draft the initial CritCom measure, the expert panel participated in a series of working meetings to identify and define independent domains of interprofessional communication quality. They then reviewed all question items that had been extracted during literature review of existing published tools and mapped these to the identified domains. This was done through group discussion and electronic survey to reach consensus. Because of the extent of prior work and already established items identified during literature review, concept mapping was not used to draft items for this measure. The survey asked individuals to rate each potential survey item on importance and clarity. Following this, the expert panel met and reviewed the results, choosing the items that were rated as the most important and clear to include in the initial measure. Items were removed for a variety of reasons including concerns about clarity, importance, and to delete duplicate concepts. Ultimately, this process resulted in a draft measure that underwent iterative rounds of review by listed collaborators to ensure both content validity and cultural sensitivity.

Step 3: cognitive interviewing (first instrument language-English)

Cognitive interviewing is an established method to assist with identification of problematic survey items while establishing face validity (28). Participants are asked to both complete the draft measure and answer questions about how they interpreted items, what they found difficult to answer, and what concepts they think the items are capturing (29). Cognitive interviewing is typically done via semi-structured interviews with a representative sample of future users of the tool.

Since the goal of developing CritCom was to administer it in English and Spanish, we first completed cognitive interviewing in English, followed by translation, then repeated the same process in Spanish (see Step 5). We consider this a best practice for two reasons: first, this ensures clarity in both languages and second, this reduces the potential for conflicting edits between languages. For our English-language cognitive interviewing, the sampled target participants included English-speaking intensive care unit and ward nurses and physicians from centers located in different countries and various resource levels.

A standard semi structured cognitive interview guide was developed (Supplementary Material S1) and updated with each round of interviewing. These interviews were virtually conducted over Zoom (by SM, KP, JR) with audio and cameras enabled to facilitate the interview. This allowed for better simulation of in-person interviews and improved communication. Participants were interviewed in phases of 3–5 interviews at a time, after which the core team (JR, SM, AsA, MPT, KP) met to adjust both the measure and the interview guide. Feedback by participants was used to adjust CritCom wording to optimize clarity and ensure appropriate comprehension of each survey item. A total of 19 English-speaking participants from 9 countries were interviewed (Table 2). This included native and non-native English speakers, as we anticipated that many participants taking the survey in English would not be native English speakers. This process allowed for iterative revisions of the measure to ensure comprehension and face validity. Interviews were stopped when no further changes were needed for the English version of the initial items.

Step 4: translation

A formal, thorough translation process is needed for measures intended to be delivered in multiple languages. Using forward-backward translation is a standard approach (30). This process involves translation to the second language, and then translation back to the original language by a different individual or group to compare with the original version. However, even within languages there are regional and dialect differences impacting the interpretation of survey items across and within different countries and regions. Appropriate attention must be paid to these differences and their potential implications for item interpretation and construct definitions. To best address these, our method emphasizes using translators with different dialects from different regions speaking the same language, and then reconciling language differences to achieve linguistic validity.

In our study, survey questions were first translated from English to Spanish through a professional translation company, then iteratively reviewed by five bilingual members of the research team (JR, MPT, HMT, AnA, AsA) from five different countries (Mexico, Puerto Rico, Chile, Colombia, USA). Collaborators reviewed the translation for colloquial syntax, comprehension, cultural relevance, and questions were modified as needed to enhance cultural and linguistic validity. This process allowed the team to account for regional differences and resolve them in a way that was most generalizable to all Spanish speakers regardless of origin.

Developing conceptually equivalent surveys is challenging. We faced challenges related to regionalism, education background, preferences for more or less formal phrases, and English words/terms for which there is no exact Spanish equivalent (e.g., “speak up” and “actionable”). We selected the final translations to maximize: (i) the consistency of the Spanish translation with the intent of the English version; (ii) clarity; and (iii) similarities in understanding between regions (31).

Step 5: cognitive interviewing (second instrument language-spanish)

As described above, cognitive interviewing ensures that the measure is understandable to participants in each language. This must be done in every language to identify items that may be problematic in that language. These steps should be repeated as necessary for all desired language-versions for measure co-development.

A second round of cognitive interviewing was repeated using the Spanish version of CritCom with native Spanish-speaking participants, using a similar process as described in Step 3. The English-language cognitive interview script was translated into Spanish using a similar process as described above; specific questions were added to target survey items that were challenging to translate in Step 4. Bilingual, native Spanish speaking members of the research team (JR, MPT) conducted cognitive interviews with participants from 6 Spanish-speaking countries from Mexico, Central and South America, representing the same target participant demographic as Step 3. Interviews were stopped after 17 participants, when saturation was reached and no further changes were recommended. Like in Step 3, interviews were conducted in phases. After each round of 3–5 interviews, the core team met (JR, SM, AsA, MPT, KP) to review feedback and edit the tool as needed. When change was made to the measure, back-translation to English was done to ensure that the intent of the questions remained intact. Input on this process was provided by native Spanish and English speakers to ensure that final questions were appropriate in both languages.

Step 6. Language Synthesis

A final review of the items must be completed to confirm that the items are still the same across languages. A unique benefit of this co-design process is the ability to use the second language (Spanish) to confirm face validity and further refine the original English language measure.

Translation to and cognitive interviewing with the Spanish-language measure identified additional wording that was unclear in the English tool and both measures were adjusted based on feedback (e.g., speak up vs. “alzar la voz”; speak up is translated as “raise your voice” in Spanish, which is not the intended meaning and would represent a completely different interpretation). This opportunity for synthesis between feedback derived from evaluating both language-versions is a unique benefit of co-development of multilingual measures.

Step 7: final review

Following the described structured method for development including cognitive interviewing and synthesis of feedback, the bilingual measure confirmed content, face, and linguistic validity. In a final step, the English and Spanish versions of the instrument were reviewed by the entire expert panel. No further edits were made to the instrument at this step; this review served as a final confirmation that the two versions were identical, clear to all individuals, and achieved the initial objectives for this measure. To ensure measure reliability and validity, further psychometric testing is required. However, this provides a rigorously developed draft measure for pilot testing.

## Critcom development and results

**Figure 2:**
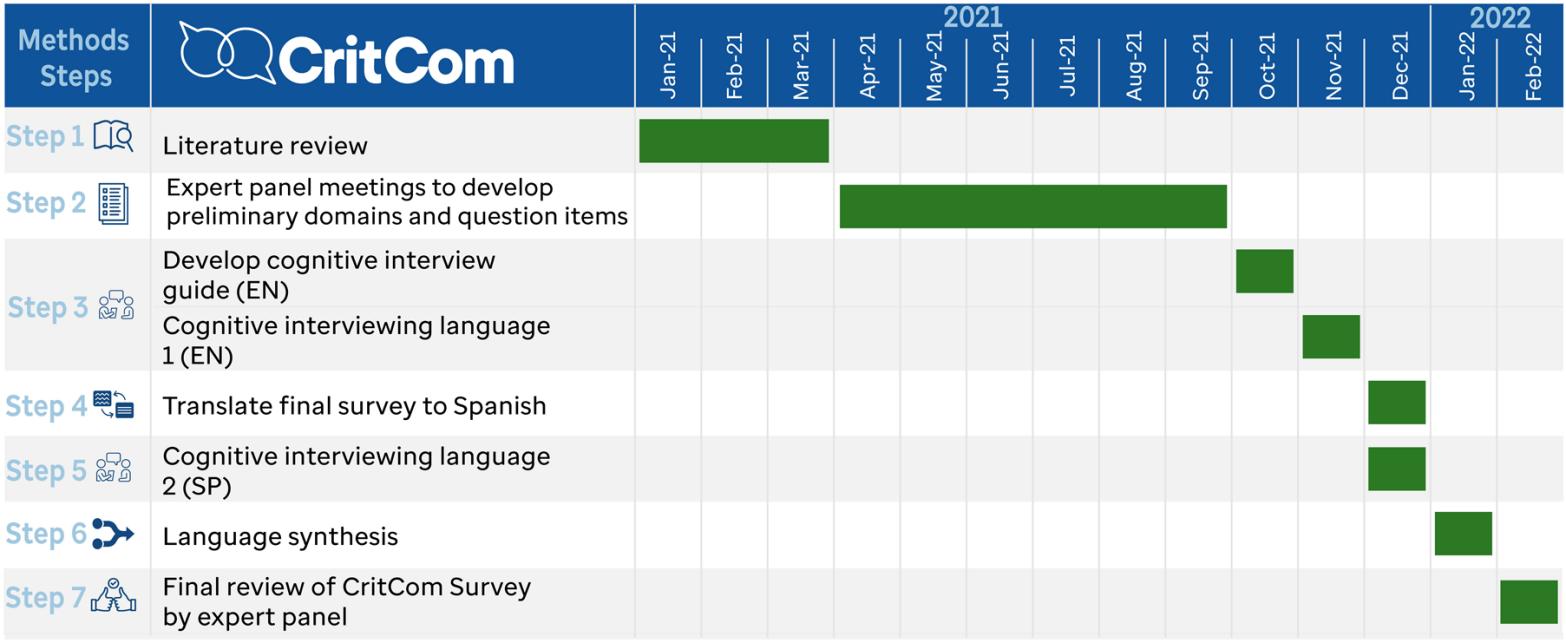
Timeline for CritCom development.

**Figure 3:**
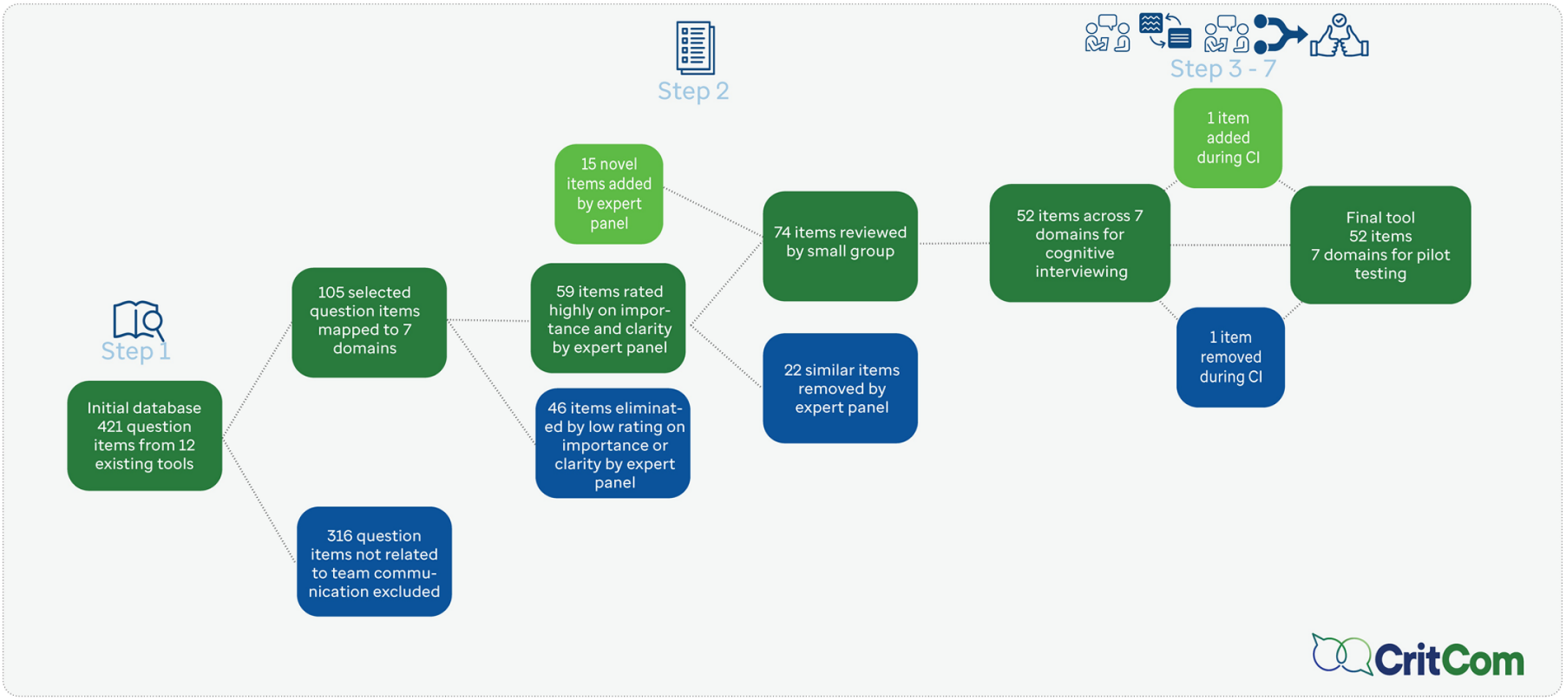
Flow diagram of CritCom items.

Figure 2 presents the steps as used for CritCom with a timeline for the process and Figure 3 highlights how questions were added and dropped from the measure at each step.

### Initial measure (Steps 1–2)

The literature review (Step 1) resulted in 421 questions from 10 unique previously developed measures in related areas. After initial review by the expert panel (Step 2), 105 questions related to relevant domains of interprofessional communication were selected. Expert panel members were asked to identify the most important and clear items for the assessment, resulting in 59 items that were further reviewed for importance and clarity. After removing low-rated items, the draft instrument had 52 items across 7 domains (Table 4). The domains highlight the content of communication, the style in which it was delivered, and aspects about the team and organizational context that promote or hinder high quality communication. The items were all phrased to inquire, on a Likert scale from almost never to almost always, a clinician's perception of different aspects of communication in their unit. For example, one item under actionable communication asked if during shift changes, staff exchange all essential patient information.”

**Table 4:** Selected preliminary domains and definitions.

Domain	Number of items	Definition
Actionable	6	Communication that allows the team to get the job done. Communication is relevant, complete, timely, and contains the necessary information to act.
Clarity	6	Communication that allows for the content to be clear, structured, and to communicate a shared mental model.
Tone	7	Communication manner, including wording, non-verbal communication, and being ignored.
Mechanisms and modes	7	Structural elements of communication, including how we communicate, with whom, language barriers, and technology facilitated communication.
Collaboration and teamwork	9	Communication that allows for interdisciplinary collaboration between staff. This includes working collaboratively, teamwork, role clarity, and mutual respect.
Systems	10	Systemic elements that improve or impede communication, including system structure, culture, reporting structures, and hierarchy.
Empowerment	7	Communication that allows team members to proactively evaluate patients, make decisions, speak up, and escalate concerns without fear of consequences.

### Cognitive interviews and translation (Steps 3–6)

A total of 36 English and Spanish-speaking individuals from 15 countries were interviewed during this process (Table 2). This included 25 doctors and 11 nurses from both Pediatric Hematology-Oncology and Critical Care programs. Cognitive interviews were conducted in rounds, where 2–4 individuals were interviewed prior to the research team reviewing interview notes and making edits to the tool. The English interviews were conducted in 8 rounds, with edits made after each round except the last. The Spanish interviews were conducted in 5 rounds, with edits being made in a similar fashion. All interviews were conducted in individual's native language and were recorded on Zoom, with interviews lasting 15–60 min. Participants were able to identify language and content changes needed to improve the measure (Table 5). As described above, following the Spanish cognitive interviews, feedback in both languages was synthesized (Step 6).

**Table 5:** Examples of items refined during cognitive interview process.

Original Item	Cognitive Interview Notes	Revised Item
Staff follows a structured method when communicating a patient concern to another team member. (EN)	Asked for clarity or example of “structured method of communication” – RN Noted that the provided example (SBAR) was helpful – Dr.	Staff follows a structured method (e.g., SBAR, IPASS, or other institutional format) when communicating a patient concern to another team member.
Feedback between staff is delivered in a way that promotes positive interactions. (EN)	understood feedback to be “results from a test or response to a question” – Dr.	Constructive criticism between staff is delivered in a way that promotes positive interactions.
Después de dialogar sobre un paciente con deterioro, el personal tiene el mismo conocimiento sobre los pasos a seguir en el manejo. (SP)	No puede decirse que se tiene el mismo conocimiento. – MD Más que el mismo conocimiento, el equipo completo debe tener los objetivos. – RN	Después de dialogar sobre un paciente con deterioro, el personal tiene el mismo entendimiento acerca de los pasos a seguir en el manejo.
Existen barreras de lenguaje entre los miembros del equipo de atención médica. (SP)	En latinoamerica, es más compun que haya diferentes dialectos incluso en un mismo país. – RN	Existen barreras de lenguaje (ej. diferentes idiomas o dialectos) entre los miembros del equipo de atención médica.

### Draft measure (Step 7)

After the final round of cognitive interviewing and language synthesis, the measure had 52 items across 7 domains in the draft CritCom instrument. This measure was reviewed by the expert panel (Step 7) resulting in no further changes. This resulted in a measure with content, face, and linguistic validity to evaluate aspects of quality communication among healthcare professionals from the perspective of in both English and Spanish speaking individuals practicing in hospitals with a range of resource-levels. This measure is now ready to pilot to remove poor performing items and evaluate the proposed domain structure through psychometric testing.

## Discussion

This paper details a novel method for simultaneous measurement co-development in multiple languages, resulting in a draft measure that has content, face, and linguistic validity. We accomplished this through engagement of interdisciplinary, multi-professional experts in clinical care, measurement development, and communication. The 7-step process described is both rigorous and inclusive while maintaining efficiency, and it advances measurement development in two important ways. First, this method ensures that created measures have strong face and linguistic validity and that they are pragmatic for researchers and practitioners in a variety of practices and linguistic settings. Second, this method addresses concerns of health equity in implementation science (32, 33) by facilitating development of multilingual measures that can be utilized across a variety of participant populations, including those who have been previously excluded due to language barriers.

Measure development using this method requires broad considerations about the target populations and settings. To assure linguistic validity, special considerations must be given to the variety of dialects and regional variation that may influence the translation and comprehension of the new measure. Additionally, phrasing of related components that may be difficult to interpret for participants answering the survey in a non-native language (as is common in global health research), such as the instructions, must be considered. Finally, measures that are intended for a variety of audiences must consider the literacy levels and experiences of all individuals who may complete the measure. These challenges are best assessed through cognitive interviewing that is inclusive and comprehensive of the target participant population discipline, profession, and language (34–36). Measure development without these considerations can have potential problems, including inaccurate measurement of a construct and systematic misrepresentation of specific participant groups.

This process, while useful, also has a few challenges that must be considered. The proposed method requires a multilingual team, including individuals from different regional settings. To conduct the cognitive interviews, it is necessary to engage a network of willing participants, which can only be fostered through appropriate community engagement in research. Intentional engagement is especially important for expanding implementation science in a global health context and emphasizing inclusion beyond individuals in high income countries (1). Committing to this process is also relatively time intensive, which means that it must be planned for and considered well before a final measure is needed.

Even after considering these challenges, we believe there are several best practices described in this 7-step process that promote rigorous methodology for measurement development in implementation science. First, translation, review, and back-translation should be conducted by multiple bilingual native speakers to reconcile regional differences in phrasing and context. Additionally, cognitive interviewing should be conducted serially and *prior* to pilot testing a new measure. This allows improvements in any language to be incorporated before any psychometric testing. Additionally, this process allows for problematic items to be identified and improved in all versions of the measure, with each language informing the others. Discussions about cultural adaptation occur during this process, thereby avoiding the need for multiple future revisions (37). The synthesis step used to align feedback between languages facilitates development of a more robust and useful measure due to the need to clearly articulate concepts in both languages. Components of the co-development process can also be applied for rigorous translation of existing measures, for which standardized guidance does not exist in the literature.

## Next steps

Following the process described, the CritCom measure will undergo psychometric testing, focusing on evaluating the 7-domain structure and shortening through removal of poorly performing items via reliability assessment and confirmatory factor analysis. This will be done through administering the survey to a group of participants, collecting data, and conducting quantitate analyses concurrently in English and Spanish. Ultimately, this process will result in a measure that can reliably and validly assess the quality of interprofessional communication with a focus on pediatric cancer settings. We expect CritCom to be ultimately used in future research to understand how team communication is implicated in the system of healthcare delivery, both as a determinant for implementation and sustainability and as an outcome to measure the impact of interventions on team communication within complex clinical systems.

## Conclusions

We described a 7-step stakeholder-engaged process which results in the development of measures with content, face, and linguistic validity and allows researchers and practitioners to measure clearly articulated and defined constructs in multiple languages. This methodology can inform the development of a broad range of measures, responding to concerns regarding the need for more rigorous measure development in implementation science. This work also responds to an important need in global health for tools that can be administered in multilingual contexts. Finally, this approach highlights how measures can be pragmatically developed for use across a wide range of settings, promoting inclusivity and equity in implementation science.

## Data Availability

The original contributions presented in the study are included in the article/**Supplementary Material**, further inquiries can be directed to the corresponding author.
