# Supplementary material for: A new measure for multi-professional medical team communication: design and methodology for multilingual measurement development
Source: Front Pediatr. 2023 Jun 2;11:1127633. doi: 10.3389/fped.2023.1127633 (PMC10272604; doi:10.3389/fped.2023.1127633)
Supplement: Supplementary file 1 [file Table1.docx]

Introductions

Thank you for agreeing to be part of this study.

I will let you know about the project and the objectives of this interview

We have developed a survey about interdisciplinary communication called “CritCom” that we plan to use with clinical staff who care for children with cancer in the hospital setting.

Before we start using it, we are getting feedback from clinicians like you, to make sure that the questions make sense and that they ask about the things that are important to you.

For this interview Im going to ask you go to the survey with a link that I will share on the chat, and I will need you to share your screen while you answer it.

The survey is dived in 7 domains, and it has 50 questions. I will ask you to stop at the end of every part so we can discuss about the questions that you just answered.

As a reminder, your participation is voluntary, and we can stop this interview at any time.

I will be recording so I can go back to review your comments.

Do you have any questions before we continue?

First, I am going to ask you about some general terms that we use in the survey.

When thinking of these terms, consider the context of the clinical care you provide as part of your job in the hospital.

- What does “good interdisciplinary communication” in the clinical setting mean to you?
- Who is included in the “interdisciplinary team”?

I will now have you take the survey in Qualtrics, section by section. After each section, we will ask you questions about what you were thinking about when you answered the questions.

Please go to this link <https://stjudeglobal.co1.qualtrics.com/jfe/form/SV_dbCxq0mgGSX56vQ>

(drop link in chat) and share your screen.

This first page is related to description, instructions, and some definitions. Please let me know if there is anything that needs to be clarified.

For every part of the survey, I will ask you to let me know if there were any questions difficult to answer or understand or if any of those sound repetitive to you.

Go ahead and complete the first part of the survey, and then I will ask you some questions.

**[Read intro text and complete Actionable items]** Now, let’s look at these questions again. These questions ask about whether communication is actionable.

- What does “actionable” communication mean to you?
  - Were any components of “actionable communication” not asked in this section?
- Q1-2 What does the word “timely” mean to you?
- Q4 What does the word “relevant” mean to you?
- Q4 What does the phrase “patient status change” meant to you?
- Q5 What does the term “shift change” mean to you?
- Q6 What does the word “complete” mean to you?

Now let’s move on to the next section. **[complete Clarity items]** Now, let’s look at these questions again. These questions ask about whether communication is clear.

- What does “clear” communication mean to you?
  - Were any components of “clear communication” not asked in this section?
- Q7 What do you think of when you hear about “structured method of communication”?
- Q8 What do you think of when you hear the phrase “standard language to communicate”?

Now let’s move on to the next section. **[complete Tone items]** Now, let’s look at these questions again. These questions ask about the tone of communication.

- What does communication “tone” mean to you?
  - Were any components of “communication tone” not asked in this section?
- Q15 What does the term “active listening” mean to you?
- Q16 What does the term “non-verbal communication” mean to you?
  - What is the right word for the type of “non-verbal communication” staff SHOULD use? Approachable? Acceptable? Non-threatening? Appropriate?

Now let’s move on to the next section. **[complete Mechanisms and Modes items]** Now, let’s look at these questions again. These questions ask about how we communicate.

- What do you think is included in the “mechanisms and modes” of communication?
  - Were any components of how we communicate not asked in this section?
- Q20 What does the phrase “standard protocol to guide escalation of care” mean to you?
- Q23 What type of “electronic communication” does your team use to communicate?
- Q24 What does “in-person” mean to you?
- Q25 What type of language barriers might a clinical team have?

Now let’s move on to the next section. **[complete Empowerment items]** Now, let’s look at these questions again. These questions ask about staff empowerment.

- What does it mean to feel “empowered” in the context of clinical care?
  - Were any components of “empowerment” not asked in this section?
- Q27 What does the phrase “speak up” mean to you?
- Q29 What does it mean to “advocate” for a care plan?

Now let’s move on to the next section. **[complete Collaboration and Teamwork items]** Now, let’s look at these questions again. These questions ask about collaboration and teamwork.

- What does “collaboration and teamwork” mean to you?
  - Were any components of “collaboration and teamwork” not asked in this section?
- Q17 What does the word “feedback” mean to you?
- Q38 What does the phrase “constructive criticism” mean to you?
- Are questions 40 and 41 asking about different groups of people?
  - Which better reflects teamwork in your unit?

Now let’s move on to the next section. **[complete Systems items]** Now, let’s look at these questions again. These questions ask about hospital systems and communication.

- What does the term “systems” mean to you in the context of communication?
  - Were any components of “systems” not asked in this section?
- Q 41-44 What does the word “unit leadership” mean to you? Who did you think of when answering these questions?
- Q 45 What does the phrase “chain of command” mean to you?
- Q 46-48 What does it mean to be “approachable”?
- Q 49 What does the word “hierarchy” mean to you? How is this different from chain of command?
- Q50 What is an example of a person acting in a way that “impedes effective communication”?

Now let’s move on to the next section. **[complete Demographic items]** Now, let’s look at these questions again. These questions ask about your role and background.

- Were there any questions that you found difficult to answer or understand?

Now, let’s consider the survey as a whole.

- Overall, how do you feel answering these questions?
- The survey will be delivered anonymously. Do you feel you can be honest? Do you feel that the rest of the staff could be honest about their answers?
- How did you feel about the length of the survey?
- Did these questions allow you to describe how the clinical team in your unit communicates? Are there aspects of communication that we did not address?
- You may note that we used phrases such as “your team”, “your unit”, “staff”, and “patient deterioration” throughout the survey. Did you have trouble interpreting these phrases? Are there better terms to use?
  - Were there other commonly used phrases that you had trouble interpreting, that would benefit from definitions?
- Some questions ask about your experience, and others ask about your team and other staff. Did you have trouble answering what typically happens in your unit?
- Related to the options that we gave (Likert scale):
  - Do you think the option for “not applicable” is necessary?
  - Would you prefer a 7-point likert scale to the 5-point we have currently (never to always)?
- Are there other things you would like us to know about interdisciplinary communication in your hospital?
- Do you have other comments about the survey that you’d like for us to know?

Thank you again for being so generous with your time. We appreciate your feedback.
